# Massive digital gene expression analysis reveals different predictive profiles for immune checkpoint inhibitor therapy between adenocarcinoma and squamous cell carcinoma of advanced lung cancer

**DOI:** 10.1186/s12885-022-09264-2

**Published:** 2022-02-08

**Authors:** Toshihiko Kaneda, Takayasu Kurata, Tomoko Yoshida, Kayoko Kibata, Hiroshige Yoshioka, Hiroaki Yanagimoto, Kazuhiko Takeda, Takao Yoshida, Koji Tsuta

**Affiliations:** 1 Department of Thoracic Oncology, Kansai Medical University Hospital, 2-3-1, Shinmachi, Hirakata City, Osaka 573-1191 Japan; 2 Discovery Technology Research, Ono Pharmaceutical, Co., Ltd., Osaka, Japan; 3 Department of Surgery, Division of Hepato-Biliary-Pancreatic Surgery, Kobe University Graduate School of Medicine, Kobe, Japan; 4 Corporate Sponsored Research Programs for Cancer Immunogenomics, Kansai Medical University, Osaka, Japan; 5 Research Center of Oncology, Ono Pharmaceutical, Co., Ltd., Osaka, Japan; 6 Department of Pathology, Kansai Medical University, Osaka, Japan

**Keywords:** Advanced non-small cell lung cancer, Nivolumab, Anti PD-1 antibody, Transcriptome

## Abstract

**Background:**

Immune checkpoint inhibitors prolong the survival of non-small cell lung cancer (NSCLC) patients. Although it has been acknowledged that there is some correlation between the efficacy of anti-programmed cell death-1 (PD-1) antibody therapy and immunohistochemical analysis, this technique is not yet considered foolproof for predicting a favorable outcome of PD-1 antibody therapy. We aimed to predict the efficacy of nivolumab based on a comprehensive analysis of RNA expression at the gene level in advanced NSCLC.

**Methods:**

This was a retrospective study on patients with NSCLC who were administered nivolumab at the Kansai Medical University Hospital. To identify genes associated with response to anti-PD-1 antibodies, we grouped patients into responders (complete and partial response) and non-responders (stable and progressive disease) to nivolumab therapy. Significant genes were then identified for these groups using Welch’s t-test.

**Results:**

Among 42 analyzed cases (20 adenocarcinomas and 22 squamous cell carcinomas), enhanced expression of *MAGE-A4*, *BBC3,* and *OTOA* genes was observed in responders with adenocarcinoma, and enhanced expression of *DAB2*, *HLA-DPB,1* and *CDH2* genes was observed in responders with squamous cell carcinoma.

**Conclusions:**

This study predicted the efficacy of nivolumab based on a comprehensive analysis of mRNA expression at the gene level in advanced NSCLC. We also revealed different gene expression patterns as predictors of the effectiveness of anti PD-1 antibody therapy in adenocarcinoma and squamous cell carcinoma.

## Background

Approximately 80% of lung cancers are non-small cell lung cancers (NSCLCs) [1]. Although surgical resection is the most effective therapy for early-stage NSCLC, up to two-thirds of NSCLCs are inoperable. This implies that histological and molecular diagnosis should be established using tiny specimens, such as small biopsy specimens and/or cytological materials, obtained via transbronchial biopsy, fine-needle aspiration or computed tomography (CT) guided biopsy. With the advent of molecular targeted therapies for NSCLCs, specimen requirements continue to increase [2].

Since the discovery of the association between epidermal growth factor receptor (*EGFR*) mutations in NSCLCs and their tyrosine kinase inhibitors, various gene alterations have been identified, and therapeutic agents based on these alterations have shown dramatic effects [3]. A portion of patients without druggable driver oncogenic alterations show dramatic durable responses to immune checkpoint inhibitors (ICIs) [4, 5]. On the contrary, ICI therapy sometimes shows serious side effects such as autoimmune diseases. Therefore, appropriate patient selection is imperative [6].

The most commonly used predictor of ICI efficacy is immunostaining for programmed cell death-ligand 1 (PD-L1) protein. Even in patients with high PD-L1 protein expression, the accuracy of prediction is reported to be only approximately 50%. In addition, some patients without PD-L1 protein expression have shown responses to ICIs [7]. Furthermore, there is significant intratumoral, intertumoral, and temporal heterogeneity in PD-L1 protein expression, which can lead to misclassification of PD-L1 status [8]. Some researchers have explored other predictive biomarkers for ICIs. Tumor mutation burden is a useful biomarker candidate [9], but its detection requires genetic analysis by next-generation sequencing. The tumor microenvironment is also considered a seminal predictor of the efficacy of ICIs.

Comprehensive mRNA profiling is now used to predict the therapeutic effects of ICIs and prognosis of malignancies. In fact, comprehensive gene analysis techniques, such as NanoString’s Prosigna™ Breast Cancer Prognostic Gene Signature Assay, are used in daily clinical practice for patients with breast carcinoma and play an important role in therapeutic decisions [10]. The NanoString’s platform hybridizes fluorescent barcodes directly to specific nucleic acid sequences, allowing for the non-amplified measurement of up to 800 targets within one sample.

The aim of this study was to retrospectively explore mRNA profiles, considered to be involved in the tumor microenvironment, as a predictor for ICIs using NanoString’s platform, and to verify whether daily cytological specimens could be effective predictors for ICIs.

## Methods

### Patients and samples

We investigated patients with NSCLC who were administered nivolumab at the Department of Thoracic Oncology, Kansai Medical University Hospital (Osaka, Japan). Nivolumab (3 mg/kg) was intravenously administered every two weeks (a cycle) unless progressive disease (PD) or unacceptable toxicity was noted. Patients were followed up at regular intervals for evaluation of recurrence. Chest CT scans were performed every two to three cycles to evaluate the treatment response and disease progression.

Patient characteristics were collected from medical and radiographic records to ascertain age, sex, smoking history, histology, *EGFR* mutation status, and PD-L1 protein expression status. Tumor response was retrospectively evaluated according to the Response Evaluation Criteria in Solid Tumors version 1.1. The efficacy of the treatment was assessed by two people: the attending physician and one of the physicians in charge of this study. Progression-free survival (PFS) duration was calculated from the date of initiation of nivolumab treatment to the date of disease progression or death. Overall survival (OS) time was determined from the date of initiation of nivolumab treatment to the date of death or last follow-up on December 31, 2018.

### RNA extraction

All cytological specimens selected for RNA extraction were those obtained from patients before nivolumab administration. A cytological slide with tumor content of 30% or more was judged to be appropriate. Slide coverslips were detached in xylene, and the slides were rehydrated by successive ethanol washes (95, 70, 50, and 30%), followed by soaking in phosphate-buffered saline for 2 min. The slides were then air-dried. Using a new, flat, single-edged razor blade, the entire contents of the slide were scraped into 200 μL of phosphate-buffered saline. Tumor enrichment with macrodissection was performed as required. RNA extraction was performed using the QIAamp RNA Blood Mini Kit (Qiagen, Germantown, MD, USA) according to the manufacturer’s protocol.

### Digital mRNA counts and analysis

The nCounter assay was performed using the NanoString nCounter mRNA Gene Expression system and nCounter® PanCancer IO 360 Panel (NanoString Technologies, Inc.) according to the manufacturer’s instructions. RNA was hybridized with probe sets for 16 h at 67 °C. The samples were processed using an automated nCounter Sample Prep Station (NanoString Technologies, Inc.). Cartridges containing immobilized and aligned reporter complexes were subsequently imaged on an nCounter Digital Analyzer (NanoString Technologies, Inc.) that had been set at a data resolution of 555 fields of view.

### Gene expression analysis

Reporter counts were subjected to sequential data processing steps using the nSolverTM Analysis Software (version 4.0) according to the manufacturer’s instructions. After quality control, background was calculated using the mean + 2 standard deviations of the internal negative control counts, and background subtraction was performed. The counts were normalized to internal positive controls to eliminate technical variability of the assay, and then normalized to the geometric mean of endogenous housekeeping genes. The normalized counts were analyzed using Genedata Profiler ver. 12.0.8.

### Statistical analysis

To identify genes associated with response to anti-programmed cell death-1 (PD-1) antibodies, we grouped patients into responders (complete response and partial response [PR]) and non-responders (stable disease and PD). PFS and OS curves were generated according to the Kaplan–Meier method using the log-rank test. Statistical analyses were performed using JMP 9 software (SAS Institute Inc., Cary, NC, USA). Significant genes were then identified for these groups using Welch’s t-test (Genedata Profiler ver. 12.0.8). Significantly high expression of mRNA was defined as fluctuated 2 times and genes satisfying *P* < 0.05. Overall, 18 genes related to tumor inflammation signature (TIS) were analyzed by unsupervised hierarchical clustering analysis using R3.6.0. Samples and genes were clustered using the Ward method based on Manhattan distance and Pearson distance, respectively.

## Results

### Patients and samples

A total of 93 patients received nivolumab between December 2015 and March 2018. Only 46 adenocarcinoma or squamous cell carcinoma cases with extra cytological slides were selected, considering the exhaustion of formalin-fixed paraffin-embedded blocks for previous companion diagnostics and specimen storage for future clinical trials. During the molecular analysis, four cases with low quantities of mRNA or abnormal signals were excluded, and the final cohort consisted of 42 cases (20 adenocarcinomas and 22 squamous cell carcinomas).

The clinical characteristics of the 42 patients are presented in Table 1. The median age was 69 years (range, 43–85 years). Most patients were male (34/42, 81.0%) and had smoked (37/42, 88.1%) at some point. Squamous cell carcinoma (22/42, 52.48%) was predominant. *EGFR* mutations were detected in two cases (2/42, 4.8%). As there is essentially no need to confirm *EGFR* mutation in squamous cell carcinoma, the *EGFR* mutation status of many of the cases (18/42, 42.8%) remains unknown. Further, during the study period (between 2015 and 2018), measurement of *ROS1* and *BRAF* mutations was not covered and required by the insurance system. Moreover, there were few residual specimens. As a result, additional confirmation of these mutations was not possible. Most patients received nivolumab as second-line chemotherapy (22/42, 52.4%), and the mean number of chemotherapy cycles was 9 (range, 1–64 cycles). However, measurement of PD-L1 was not mandatory in second-line chemotherapy with nivolumab and beyond during this study period, and there were few residual specimens, making additional confirmation impossible. As a result, the PD-L1 status was also predominantly unknown (32/42, 76.2%).

**Table 1:** Baseline clinical characteristics of all patients

Patient characteristics	All patients (*n* = 42)	%
Age (years)		
Median (range)	69 (43–85)	
< 70	23	54.8
≥70	19	45.2
Sex		
Male	34	81.0
Female	8	19.0
Smoking history		
Never	5	11.9
Current or former	37	88.1
Histology		
Adenocarcinoma	20	47.6
Squamous cell carcinoma	22	52.4
*EGFR* mutation status		
Del-19	1	2.4
Exon 18	1	2.4
Wild type	22	52.4
Unknown	18	42.8
PD-L1 (TPS)		
0	2	4.8
5–49	3	7.1
≥50	5	11.9
Unknown	32	76.2
Number of previous chemotherapy regimen		
1	22	52.4
2	8	19.0
≥3	12	28.6
Number of administrations		
Median (range)	9 (1–64)	
1–5	11	26.2
6–10	13	31.0
11–30	10	23.8
≥31	8	19.0
Type of specimen material		
Brush	29	69.1
TBB	8	19.0
FNA (Lymph node)	4	9.5
CTGB	1	2.4

*EGFR* epidermal growth factor receptor, *Del-19* 19 deletion, *PD-L1* programmed cell death ligand 1, *TPS* Tumor Proportion Score, *TBB* transbronchial biopsy, *FNA* fine-needle aspiration, *CTGB* computed tomography guided biopsy

Most of the cytological materials analyzed consisted of brush from bronchoscopy (29; 69.1%) followed by imprinting from transbronchial biopsy (8; 19%), fine needle aspiration from lymph node deposit (4; 9.5%), and imprinting from CT-guided biopsy (1; 2.4%).

### Treatment efficacy

The antitumor activity results are summarized in Table 2. The overall response rate (ORR) was 42.8%, and the overall disease control rate was 69.0%. In adenocarcinoma, 7 cases with PR were assigned to responders, and the remaining 13 cases were assigned to non-responders. In squamous cell carcinoma, 11 cases with PR were assigned to responders, and the other 11 cases were assigned to non-responders.

**Table 2:** Summary of antitumor activity

All patients (*n* = 42)	Number	Percent
Type of response		
PR	18	42.8
SD	11	26.2
PD	13	31.0
Objective response rate		42.8
Disease control rate		69.0

*PR* partial response, *SD* stable disease, *PD* progressive disease

Table 3 shows the differences in ORR due to each clinicopathological factor. There was no significant difference in response rate according to the histological type. There were two cases with *EGFR* mutations, both of which were PD. There were two PD-L1 negative cases, both of which were evaluated for PR.

**Table 3:** Summary of Overall Response Rate (ORR)

All patients (*n* = 42)	Number	Percent
Histology	42	
Adenocarcinoma		
ORR	7/20	35.0
Squamous cell carcinoma		
ORR	11/22	50.0
*p* = 0.367^a^		
*EGFR* mutation status	24	
Positive		
ORR	0/2	0
Wild-type		
ORR	8/22	36.4
*p* = 0.536^a^		
PD-L1 (TPS)	10	
≥1%		
ORR	5/8	62.5
< 1%		
ORR	2/2	100.0
*p* = 0.467^a^		

^a^Fisher’s exact test

**Fig. 1:**
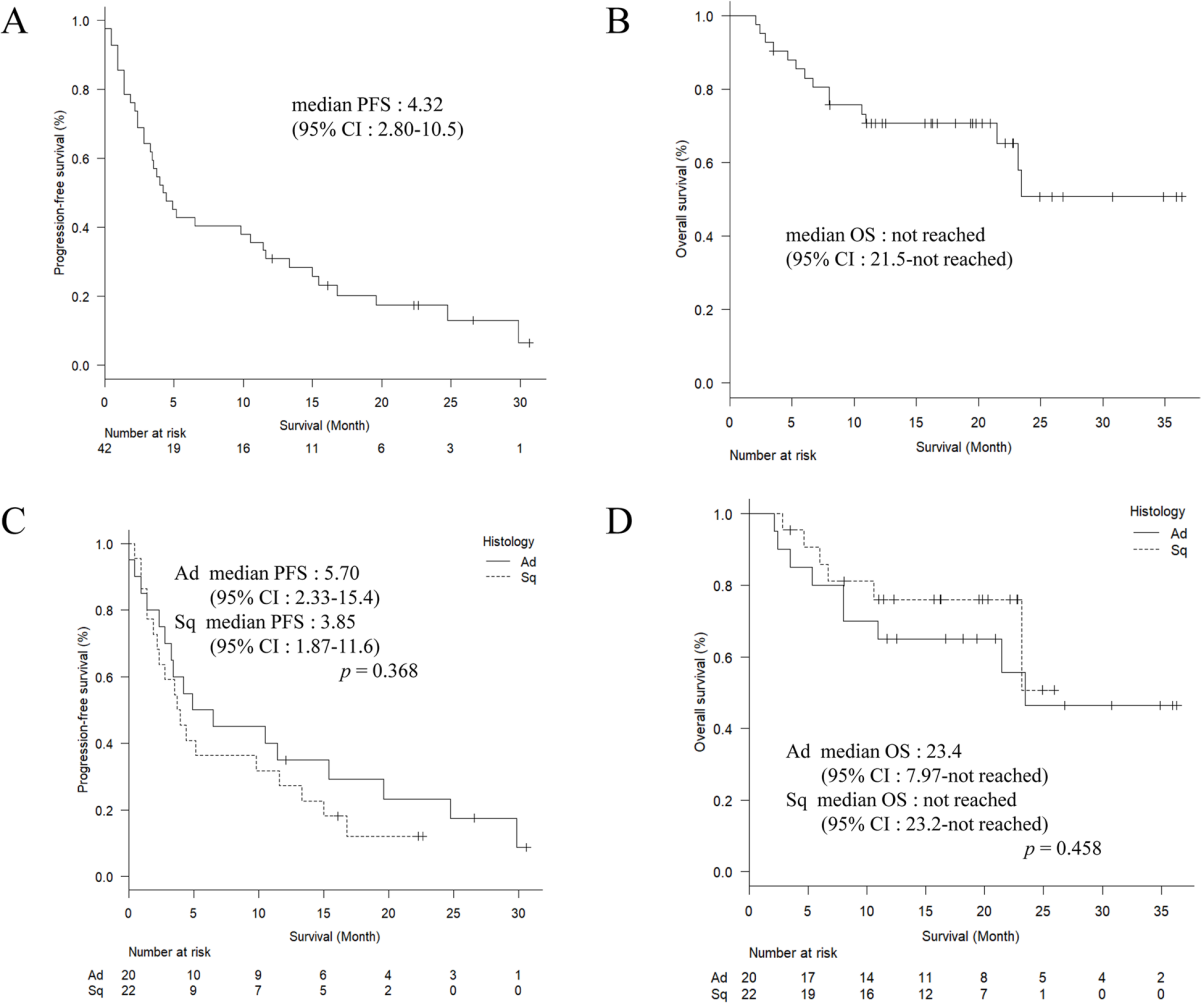
Progression-free survival (**A**) and overall survival (**B**) for all the patients. Progression-free survival (**C**) and overall survival (**D**) classified by histological type. Solid line indicates adenocarcinoma and dashed line indicates squamous cell carcinoma

In all patients, the median PFS was 4.32 months (95% confidence interval [CI]: 2.80–10.5) (Fig. 1A). The median OS was not reached (95% CI: 21.5–not reached) (Fig. 1B). There was no significant difference in median PFS between histological subtypes (adenocarcinoma; 5.70 months, 95% CI: 2.33–15.4 and squamous cell carcinoma; 3.85 months, 95% CI: 1.87–11.6, *p* = 0.386; Fig. 1C). There was no significant difference in median OS between histological subtypes (adenocarcinoma; 23.4 months, 95% CI: 7.97–not reached and squamous cell carcinoma; not reached, 95% CI: 23.2–not reached, *p* = 0.458; Fig. 1D). The median follow-up period was 16.3 months.

### Expression profiles of adenocarcinoma and squamous cell carcinoma

**Fig. 2:**
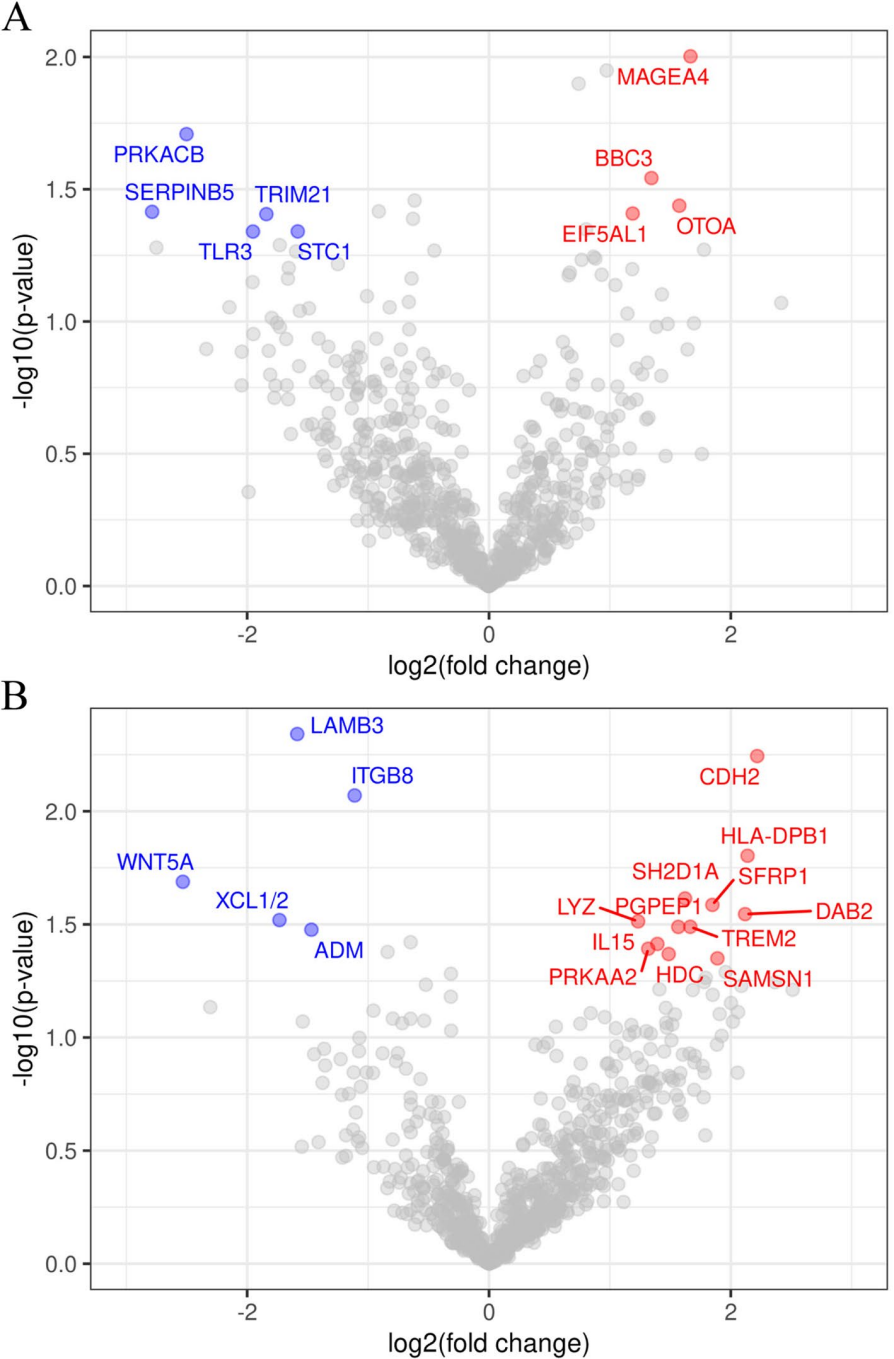
Volcano plot of unadjusted *p*-value versus log_2_ fold change of the differential expression between responders (PR) and non-responders (PD/SD) in adenocarcinoma (**A**) and squamous cell carcinoma cohort (**B**). The test for differential expression was performed using Welch’s t-test. Dots corresponding to genes with *P* < 0.05 and with expression 2-fold higher than those in non-responders are labeled in red, and those with *P* < 0.05 and expression 2-fold higher than those in responders are labeled in blue

A volcano plot of *p*-value versus log2 fold change of the differential expression between responders and non-responders in adenocarcinoma revealed that the numbers of genes with high expression in responders and non-responders were four and five, respectively (Fig. 2A). In squamous cell carcinoma cases, the corresponding numbers were 12 and 5 (Fig. 2B).

**Fig. 3:**
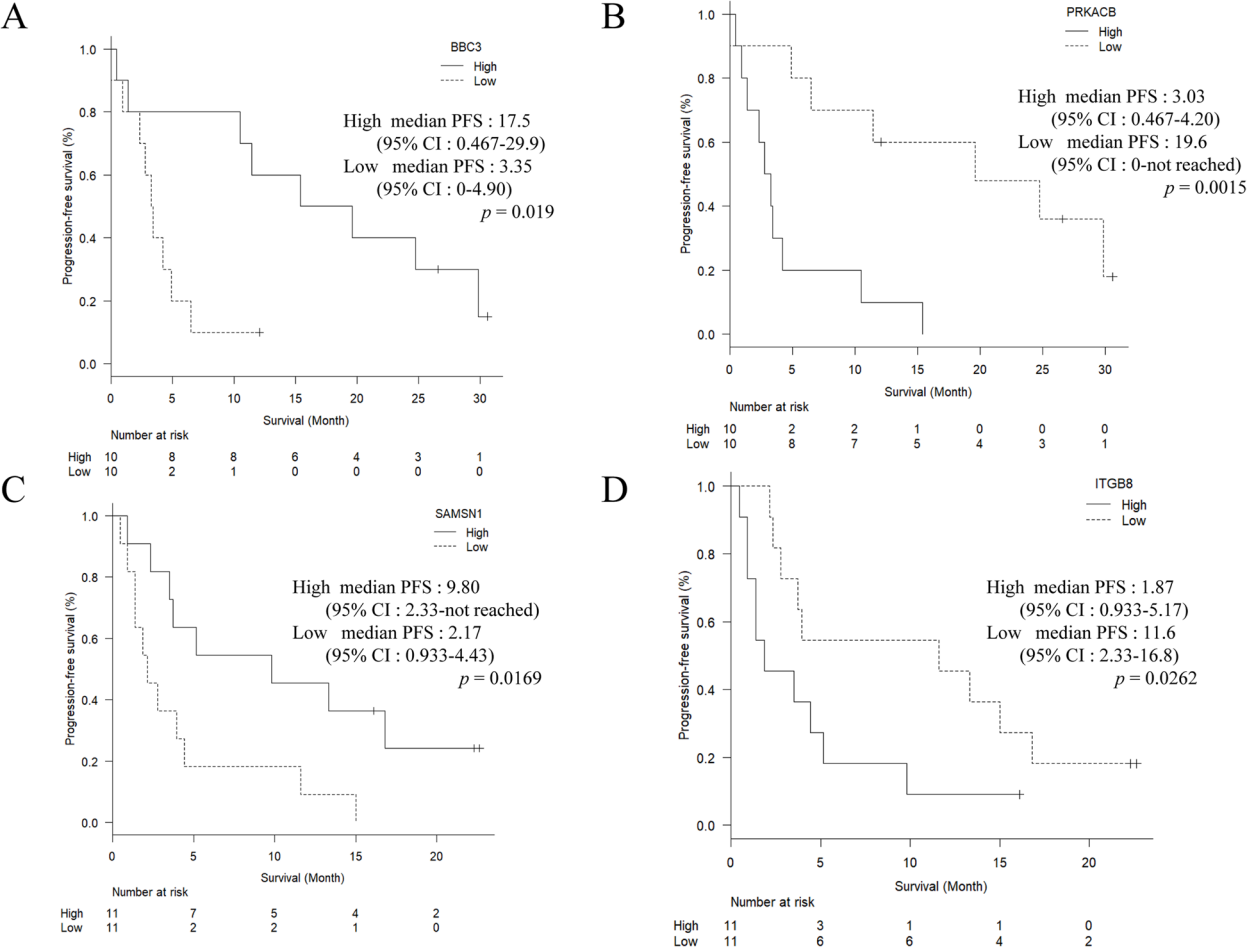
Association between gene expression profiles and progression-free survival. Kaplan-Meier curves analyses for patients with high *BBC3* expression versus low *BBC3* expression; which showed with responders in adenocarcinoma (**A**). Kaplan-Meier curves analyses for patients with high *PRKACB* expression versus low *PRKACB* expression; which showed with non-respondesr in adenocarcinoma (**B**). Kaplan-Meier curves analyses for patients with high *SAMSN1* expression versus low *SAMSN1* expression; which showed with responder in squamous cell carcinoma (**C**). Kaplan-Meier curves analyses for patients with high *ITGB8* expression versus low *ITGB8* expression which; showed with non-responder in squamous cell carcinoma (**D**)

The correlation between expression genes and PFS was examined. The median values for each gene were defined as the cutoff values separating the high and low gene expression groups. The PFS of the patients with high *BBC3* expression was longer than that of the patients with low *BBC3* expression; which showed with responders in adenocarcinoma (Fig. 3A). The median PFS of the patients with high and low *BBC3* expressions was 17.5 months (95% CI: 0.467–29.9) and 3.35 months (95% CI: 0–4.90), respectively (*p* = 0.0109). The PFS of the patients with low *PRKACB* expression was longer than that of the patients with high *PRKACB* expression; which showed with non-responders in adenocarcinoma (Fig. 3B). The median PFS of the patients with low and high *PRKACB* expressions was 19.6 months (95% CI: 0–not reached) and 3.03 months (95% CI: 0.467–4.20), respectively (*p* = 0.0015). The PFS of the patients with high *SAMSN1* expression was longer than that of the patients with low *SAMSN1* expression; which showed with responders in squamous cell carcinoma (Fig. 3C). The median PFS of the patients with high and low *SAMSN1* expressions was 9.80 months (95% CI: 2.33–not reached) and 2.17 months (95% CI: 0.933–4.43), respectively (*p* = 0.0169). The PFS of the patients with low *ITGB8* expression was longer than that of the patients with high *ITGB8* expression; which showed with non-responders in squamous cell carcinoma (Fig. 3D). The median PFS of the patients with low and high *ITGB8* expressions was 11.6 months (95% CI: 2.33–16.8) and 1.87 months (95% CI: 0.933–5.17), respectively (*p* = 0.0262). These results suggest that these gene expressions in the tumor tissue might be a predictor of PFS.

### T cell–inflamed 18 gene expression profile

**Fig. 4:**
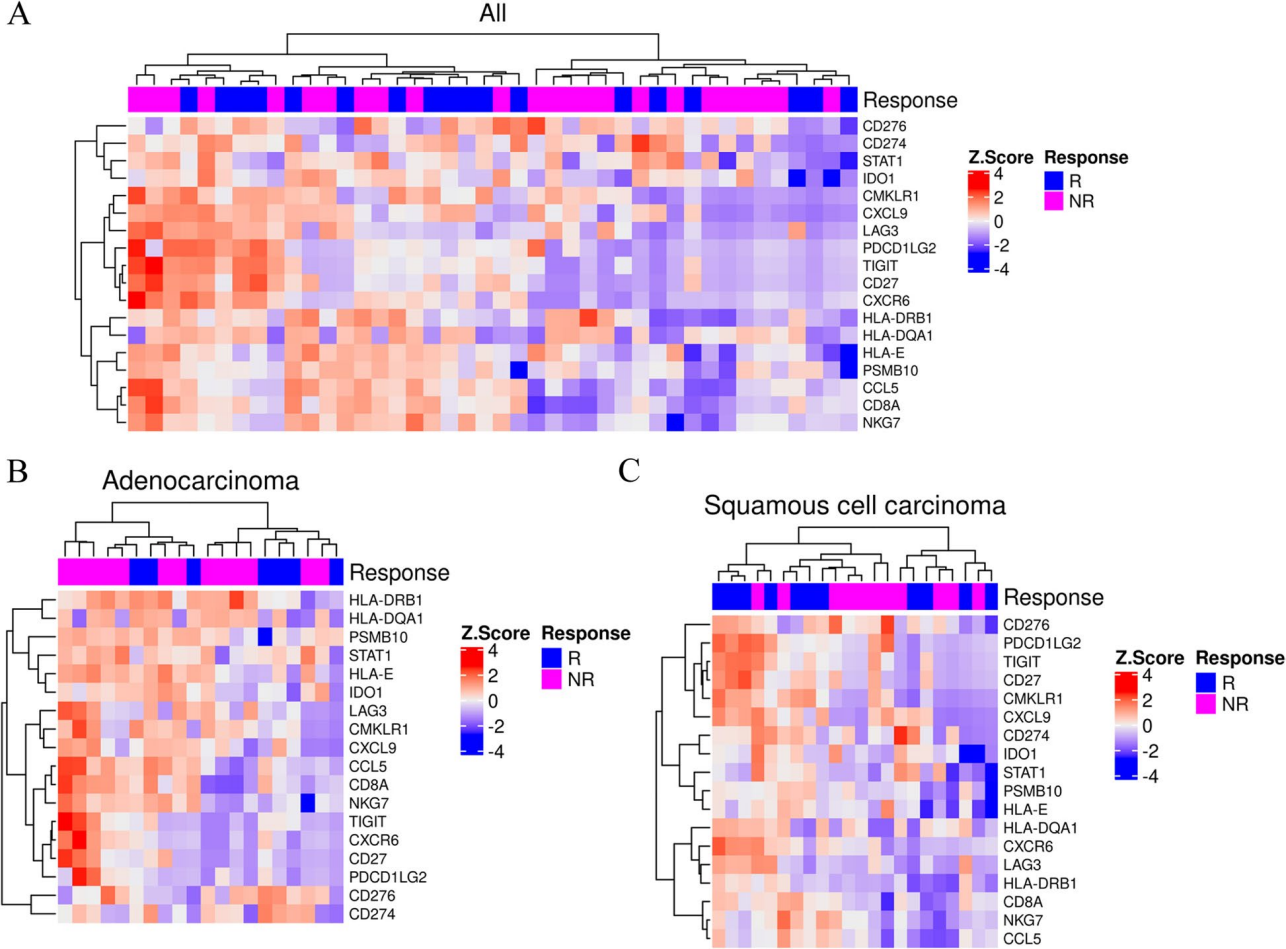
Heatmap for tumor inflammation signature (TIS) profile considered evaluable for objective response for all cases (**A**), adenocarcinoma (**B**), and squamous cell carcinoma (**C**). Columns represent patients and rows represent genes. The expression levels have been scaled within rows for visualization. The top row shows whether the patient was a responder (magenta) or a non-responder (blue)

Unsupervised hierarchical clustering analysis data between responders and non-responders are shown for all cases (Fig. 4A), adenocarcinoma (Fig. 4B), and squamous cell carcinoma (Fig. 4C). In all cases, no significant cluster formation was observed. No significant cluster formation was observed even when adenocarcinoma and squamous cell carcinoma data were separately analyzed.

## Discussion

In this study, we demonstrated that massive digital gene expression analysis can be performed using cytological specimens. We also revealed that different gene expression patterns are a predictor of the effectiveness of ICI therapy in primary lung adenocarcinoma and squamous cell carcinoma.

The results of the study show that massive digital gene expression analysis can be successfully applied to previously Papanicolaou-stained cytological materials. Although diagnostic imaging is developing, approximately 70% of lung cancer patients are detected with unresectable disease. Therefore, diagnosis must be based on small biopsy and/or cytology specimens [11]. The ‘bioresource’ used for the detection of molecular targets can be largely wasted by using this molecular approach (e.g. multiple tissue sections, dissection for tumor cell enrichment) [2]. Our result sheds light on the usefulness of cytological specimens for molecular diagnostics as well as the effective use of limited specimens [12].

We could not draw a consistent conclusion because the timing of sample collection and the timing of ICI treatment were not constant. All patients received at least one chemotherapy regimen, suggesting that those with a strong effect of cell damage from chemotherapy are more suitable for ICI therapy. Although the effectiveness of the combination of chemotherapy and ICIs has been reported [13], this mechanism may enhance it.

Among the four genes that were significantly highly expressed in the responders with adenocarcinoma, *MAGE-A4* and *BBC3* were noticeable. *MAGE-A4* has been reported to have the potential to serve as a biomarker and valuable target for immunotherapy [14]. Further, it has been reported as a target for T-cell-based immunotherapy, especially for NSCLC or triple-negative breast cancer [15], and thus it may be a potential biomarker for the therapeutic effect of ICIs. However, further data are needed.

Among the 12 genes that were significantly highly expressed in responders with squamous cell carcinoma, *DAB2* and human leukocyte antigen *HLA-DPB1* genes were related to antigen presentation and immune response, respectively. In particular, the *HLA-DPB1* gene encodes a protein that plays a critical role in the immune system. Expression of HLA molecules in cancer cells seems to be a key factor in the presentation of tumor-associated antigens to T lymphocytes [16]. Alterations of these molecules by the tumor cells are one of the important immune-escape mechanisms [17]. These facts suggest that ICIs may be more effective in cases of squamous cell carcinoma with excessive antigen presentation.

In non-responders with squamous cell carcinoma, *LAMB3, ITGB8,* and *WNT5A* genes were highly expressed. These genes are reported to be involved in tumor invasion via Rac and Rho cascades [18, 19]. Activation of the extracellular matrix and cell adhesion-related genes enhances cell motility and reduces the effect of immunotherapy. In addition, high adrenomedullin (ADM) expression was observed in the non-responder group. ADM is expressed in a variety of tissues, such as the adrenal gland, heart, and lung [20]. ADM regulates vascular tone and proliferation of fibroblasts and vascular smooth muscle cells [21]. Although there was no clinicopathological correlation, high ADM protein expression was observed in lung squamous cell carcinoma [22]. Increasing systemic levels of vasoactive peptides, including pro-ADM, are associated with improved tumor response in metastatic colorectal carcinoma patients treated with a bevacizumab-containing regimen [23]. Vascular endothelial growth factor not only promotes angiogenesis, it also causes immunosuppression in the cancer microenvironment [24]. The efficacy of combination therapy with ICIs and antiangiogenic agents has been reported [25]. This fact suggests that antiangiogenic agents enhance the effects of ICI as well as inhibit angiogenesis in a portion of squamous cell carcinomas.

Recently, the utility of a TIS consisting of 18 genes using the same NanoString platform for predicting the therapeutic effect of anti-PD-1 treatment was reported [26] and its utility has been verified in various tumors [27]. However, our cluster analysis did not confirm the utility of these 18 genes. Furthermore, the differences between responders and non-responders were evaluated by the average value of the log scores of each gene’s expression, but no statistical difference was observed (data not shown). One possible reason is that each gene is not weighted because its detailed algorithm has not been clarified. To the best of our knowledge, this study is the first to examine each histological type of adenocarcinoma and squamous cell carcinoma using a heatmap of the differential expression between responders and non-responders.

Retrospective analysis and a small sample size were limitations of the current study. Another weakness is that the time of collection and the period until drug administration were not constant. In addition, there are few cases in which known predictors, such as PD-L1 immunohistochemical status and tumor mutation burden, were analyzed; thus, an effective comparison was not possible.

Currently, the only biomarker for immune checkpoint inhibitors is PD-L1, which is not a sufficient marker in terms of accuracy. If the gene expression identified in our study can be measured in clinical practice, it could act as a new biomarker for immune checkpoint inhibitors. In addition, there is a possibility that it could be used to examine the difference between adenocarcinoma and squamous cell carcinoma, suggesting that it could act as a marker with higher accuracy than existing markers.

## Conclusions

This study aimed to predict the efficacy of nivolumab based on a comprehensive analysis of RNA expression in advanced NSCLC, finding that different gene expression patterns are predictors of the effectiveness of anti PD-1 antibody therapy in adenocarcinoma and squamous cell carcinoma.

## Data Availability

All data generated or analyzed during this study are included in the published article. All data are available from the corresponding authors upon reasonable request.

## Abbreviations

NSCLC: Non-small cell lung cancer
CT: Computed tomography
*EGFR*: Epidermal growth factor receptor
ICI: Immune checkpoint inhibitor
PD-L1: Programmed cell death-ligand 1
PD: Progressive disease
PFS: Progression-free survival
OS: Overall survival
PD-1: Programmed cell death-1
PR: Partial response
TIS: Tumor inflammation signature
ORR: Overall response rate
CI: Confidence interval
ADM: Adrenomedullin
